# Thermophilic Solid-State Anaerobic Digestion of Corn Straw, Cattle Manure, and Vegetable Waste: Effect of Temperature, Total Solid Content, and C/N Ratio

**DOI:** 10.1155/2020/8841490

**Published:** 2020-11-11

**Authors:** Lianghu Su, Xu Sun, Chenwei Liu, Rongting Ji, Guangyin Zhen, Mei Chen, Longjiang Zhang

**Affiliations:** ^1^Nanjing Institute of Environmental Sciences, Ministry of Ecology and Environment, 8 Jiangwangmiao Street, Nanjing 210042, China; ^2^School of Environmental Engineer, Nanjing Institute of Technology, No. 1 Hongjing Road, Nanjing 211167, China; ^3^Shanghai Key Lab for Urban Ecological Processes and Eco-Restoration, School of Ecological and Environmental Sciences, East China Normal University, Dongchuan Road 500, Shanghai 200241, China

## Abstract

Thermophilic solid-state anaerobic digestion (SS-AD) of agricultural wastes, i.e., corn straw, cattle manure, and vegetable waste, was carried out in this study. The effects of temperature (40-60°C), initial solid content (ISC, 17.5-32.5%), and C/N ratio (15-32 : 1) on biogas production were evaluated using a Box-Behnken experimental design (BBD) combined with response surface methodology (RSM). The results showed that optimization of process parameters is important to promote the SS-AD performance. All the factors, including interactive terms (except the ISC), were significant in the quadratic model for biogas production with SS-AD. Among the three operation parameters, the C/N ratio had the largest effect on biogas production, followed by temperature, and a maximum biogas yield of 241.4 mL gVS^−1^ could be achieved at 47.3°C, ISC = 24.81%, and C/N = 22.35. After 20 d of SS-AD, the microbial community structure under different conditions was characterized by high-throughput sequencing, showing that *Firmicutes, Bacteroidetes*, *Chloroflexi*, *Synergistetes*, and *Proteobacteria* dominated the bacterial community, and that *Firmicutes* had a competitive advantage over *Bacteroidetes* at elevated temperatures. The biogas production values and relative abundance of *OPB54* and *Bacteroidia* after 20 d of SS-AD can be fitted well using a quadratic model, implying that *OPB54* and *Bacteroidia* play important roles in the methanogenic metabolism for agricultural waste thermophilic SS-AD.

## 1. Introduction

China is facing tremendous challenges in managing its massive amount of agricultural waste in rural areas. In 2016, China produced 897 Mt of crop residues, and almost one-quarter of these crop residues were burned in the field after harvest, aggravating the air pollution [1, 2]. With the constant growth of livestock husbandry, the amount of livestock excrement in China increased by 12.8% from 367 to 414 Mt (dry) in 2007-2015, causing grave concerns about the water pollution [3].

Anaerobic digestion is a multistage biological process that, in addition to the treatment and stabilization of waste, allows the production of biogas as a versatile, renewable energy source and the recovery of residual solid (or liquid) as green fertilizers [4, 5]. Unlike other biomass resources, agricultural wastes are preferred for biogas production due to their large-scale availability and low cost, and they do not directly compete with food or feed production [6]. When compared to those of other treatments, these advantages, together with environmental impacts, make the synergistic utilization of the agricultural organic waste by anaerobic codigestion a promising technology [4].

Compared to liquid anaerobic digestion (L-AD), solid-state anaerobic digestion (SS-AD) operated at a total solid (TS) content above 15% has much higher volumetric methane productivity, requires less energy for heating and stirring, and generates less wastewater [7, 8]. The resulting residue (called digestate) of SS-AD with a lower moisture content would be favourable for transportation and can be valorized as a fertilizer for land applications [9]. Although there are many obstacles, such as difficulties in feeding and discharging, uneven mass transfer, and acid inhibition [10, 11], SS-AD has still been adopted for lignocellulosic biomass, especially in rural areas of China. In China, L-AD is facing a severe problem in treating anaerobic digester effluent, which has a high concentration of nutrients and low oxygen availability. Direct discharge of effluent to natural waters will deteriorate the water quality, affect aquatic organisms, and lead to biodiversity degradation [12]. The application of effluent to land is difficult due to insufficient storage and transportation facilities and limited land carrying capacity [13]. Further treatment to meet the threshold set by the Chinese standard for irrigation water quality (GB5084-2005) [14], such as COD ≤ 200 mg L^−1^, would be economically unfeasible.

SS-AD digesters are usually operated based on empirical knowledge rather than performance optimization [8]. The key parameters (such as moisture content, C/N ratio, and temperature) can be further optimized to achieve a better SS-AD performance. Le Hyaric et al. [15] reported that the initial substrate concentration (ISC) affects all steps of anaerobic digestion. Abbassi-Guendouz et al. [16] showed that an elevated ISC leads to lower methane production and substrate conversion. Fernandez et al. [17] observed that methane production decreased by 17% when the ISC increased from 20% to 30% in a dry mesophilic digester. Improper C/N ratios can result in high total ammonia nitrogen (TAN) release in the digester [18]. Temperature is also an extremely important factor for SS-AD; changes in temperature can alter the activity of enzymes in the microbiome and affect substrate degradation and methanogenesis [11]. It has been reported that for lignocellulosic biomass, thermophilic SS-AD led to a greater reduction in the amount of cellulose and hemicelluloses than mesophilic SS-AD [19]. However, instability is a crucial concern for applying SS-AD under thermophilic conditions [20]. Accumulation of VFAs and decreasing pH values during the start-up phase of thermophilic SS-AD digester failure have been also observed [7, 21].

To the best of our knowledge, the effects of the temperature, total solid content, and C/N ratio involved in the mesophilic SS-AD process of agricultural waste materials have been reported, but the effects of these factors on thermophilic SS-AD are poorly understood. In this study, SS-AD of corn straw, cattle manure, and vegetable waste was investigated under thermophilic conditions (40-60°C). The biogas yield, pH, and TAN concentration were characterized after 20 d of SS-AD. The effects of the temperature, ISC, and C/N ratio on biogas production were evaluated using the Box-Behnken experimental design (BBD) combined with response surface methodology (RSM). Microbial community structures were further characterized after 20 d of SS-AD using high-throughput sequencing.

## 2. Materials and Methods

### 2.1. Substrates and Inoculum

Before use, corn straw was chopped into small pieces (~1-2 cm) using a shredding machine. The cellulose, hemicellulose, and lignin contents of corn straw were 45.79%, 21.90%, and 8.01%, respectively. Cattle manure with a moisture content of 16.72% and a C/N ratio of 14.67 was obtained from a dairy farm near the city of Nanjing (Jiangsu Province, China). Vegetable waste, mainly Chinese cabbage (*Brassica rapa* L. ssp. *pekinensis*), was obtained from a local vegetable market in Nanjing. Inoculated sludge with a total solid (TS) content of 8.31% and VS content of 82.53% was collected from a methanogenic reactor that treats starch wastewater from a wastewater treatment plant (WWTP) in Shanghai, China. To reduce the endogenous gas production, the inoculated sludge was preincubated anaerobically for 7 days. Sixteen bacterial phyla were represented for inoculated sludge, with *Chloroflexi*, *Bacteroidetes*, *Proteobacteria*, and *Firmicutes* being the most abundant phyla, accounting for 33.2%, 14.7%, 11.6%, and 12.1% of the total bacterial sequences, respectively. The fundamental characteristics of corn straw, cattle manure, and vegetable waste are shown in Table 1.

### 2.2. Experimental Procedure

A three-level-three-factor Box-Behnken design (BBD) was chosen to evaluate the effects and interactive effects of the three independent variables, i.e., temperature (40°C, 50°C, and 60°C), ISC (17.5%, 25.0%, and 32.5%), and C/N ratio (15 : 1, 23.5 : 1 and 32 : 1), as *χ*^1^, *χ*^2^, and *χ*^3^, respectively, on the response variable biogas production (mL gVS^−1^) during SS-AD. The experiments were designed by Design Expert (Version 8.0.6, Statease, Minneapolis, USA), and the real values of the operating variables are summarized in Table 2. For SS-AD, corn straw, cattle manure, and vegetable waste were rigorously mixed in a mass ratio of 40 : 10 : 0.5 (dry wt.) in the laboratory. A 100 g mixed sample with a moisture content of 67.5% (adjusted by distilled water) was used as a mixed substrate. Different doses of distilled water and urea were further added to the mixed substrate to adjust the ISC and C/N ratio. Activated sludge was introduced as inoculum, with a feedstock-to-inoculum ratio (F/I, based on VS) of 3. SS-AD was carried out in 500 mL borosilicate bottles (Wente Experimental Ware, China) with a stainless steel vent pipe (Figure S1) and lasted for 20 d. Bottles were flushed with high-purity N_2_ to exclude oxygen and were sealed with a silicone gasket and stopper. The temperature during SS-AD was maintained by a thermostatic water bath (DK-98, Taisite Instrument Co. Ltd., China). An aluminium foil bag (E-switch, Shenyuan Scientific Equipment Co., China) was connected to the gas outlet by a three-way valve (Discofix, B. Braun, Germany) to collect biogas from each bottle. Different parameters, such as pH, TAN, and microbial community, were analyzed after 20 d of SS-AD. One-way ANOVA was employed to study the primary effects and interactions on biogas production between the parameters selected.

### 2.3. Analytical Methods

The pH of the digestate was measured by a digital pH meter (Sartorius, Model PB-10, Germany) after shaking for 20 min at 25°C with a liquid-solid ratio of 10 : 1. The TAN concentration of digestate extracted by ultrapure water was determined by a continuous flow analyser (Skalar San++, Netherlands). To analyze the structure of the microbial communities after 20 d of SS-AD under different operating conditions, 0.5 g of the sample was used for DNA extraction. Total DNA was extracted by a Fast DNA SPIN Kit (BIO 101, Carlsbad, CA, USA) according to the manufacturer's protocol. The V3-V4 region of the 16S rRNA gene was amplified using universal primers 338F (5′-ACT CCT ACG GGA GGC AGC AG-3′) and 806R (5′-GGACTA CHV GGG TWT CTA AT-3′). The amplicons from each sample were sequenced with the Illumina MiSeq platform (Illumina Company, San Diego, CA, USA). Diversity statistics and operational taxonomic unit (OTU) estimators were calculated using mothur software [22]. The coverage of the clone library was calculated based on the formula C = [1–(*n*1/*N*)] × 100, where *n*1 is the number of unique OTUs, and *N* is the total number of clones in a library.

## 3. Results and Discussion

### 3.1. SS-AD Performance

The cumulative biogas volumes with different parameters (ISC, T, and C/N ratio) after 20 d of SS-AD are shown in Table 3. The maximum biogas production of 236 mL gVS^−1^ was obtained at 50°C, ISC = 25%, and C/N = 23.5. It is noted that the operating parameters at 40°C, ISC = 25%, and C/N = 32 cause digester failure, generating little biogas (2.80 mL gVS^−1^) during the entire digestion process. According to the three-level-three-factor BBD, the average biogas volume yield was 110.9 mL gVS^−1^ with 12 different operating parameter settings (excluding failed digester). The results showed that optimizing the process parameters is important to promote the SS-AD performance.

### 3.2. pH and TAN Concentrations

After 20 d of anaerobic digestion, the NH_4_^+^-N concentration of each run was determined. In this study, NH_4_^+^-N was derived from cattle manure (N-rich substrates), degradation of nitrogenous organic matter (e.g., proteins and amino acids), and/or conversion of urea by deamination. TAN is a key macronutrient for microbial growth and a buffer to stabilize the pH, while high concentrations of TAN would decrease the methanogen activity and cause anaerobic digestion failure due to ammonia inhibition [23]. Production of excessive ammonia nitrogen is more likely to happen in SS-AD than L-AD due to the higher organic loading and lower water content of the former [24]. As shown in Table 3, the concentration of TAN ranged from 1641-3613 mg kg^−1^ for all samples after 20 d of anaerobic digestion. It can also be seen that a high TAN concentration was caused by a combination of elevated temperature, high ISC, and low C/N ratio. It is reported that in the L-AD system, the TAN concentration should not reach the range of 1500-3000 mg L^−1^ to avoid the toxicity of ammonia [25]. Wang et al. [24] showed that a TAN level of 4.3 g kg^−1^ caused a reduction in the reaction rates and microbial activities for hydrolysis of cellulose and methanogenesis from acetate, and a TAN level of 2.5 g kg^−1^ decreased the methane yield during SS-AD of corn stover. Therefore, it is believed that the biogas yield from runs 4, 6, and 10 was inhibited due to the excessive TAN levels (greater than 3.0 g kg^−1^). It seems that the SS-AD failure of run 7 was not caused by the accumulation of VFA (decreasing pH value) or excessive TAN level. The cause for the SS-AD failure of run7 is still unclear. TAN includes both NH_4_^+^ and free ammonia (FAN, NH_3_). FAN is the main cause of ammonia inhibition because it is membrane permeable [26, 27]. So far, a FAN calculation method has rarely been applied in SS-AD, the digestate of which generally has a higher ionic strength than that in L-AD [23]. FAN inhibition is related to the characteristics of the substrate, pH, process temperature, concentrations of ammonium and ammonia, etc. [28]. Capson-Tojo et al. [4] demonstrated that pH and temperature, rather than the TAN content itself, are the main factors affecting FAN inhibition. Among the high-TAN samples, the biogas yield of run 10, with a higher pH value, was much greater than that of run 4 and run 6. This phenomenon might be related to the higher FAN concentrations at increasing temperatures [23].

### 3.3. Modeling of SS-AD for Biogas Production

The results in Table 3 were used for multiple regression analysis using the polynomial model equation. This approach enabled the prediction of the optimum degree of biogas production and its corresponding optimum variables. One-way analysis of variance (ANOVA) was employed to test and analyze different models. In this study, the best-fit model found for the biogas production was a quadratic model. The *F* value of the model was 8.45, with a low *p* value of 0.0051. However, the *F* value (0.95) and *p* value (0.0256) of the lack of fit implied that the prediction for the fit of the model was not good. Further, studentized residual analysis showed that run 2 was an outlier due to its externally studentized residuals being larger than 5. Therefore, the data of run 2 were not included in the following modeling analysis.

The following polynomial quadratic model for biogas production was then adjusted to a better prediction by applying response surface regression (RSREG), as shown in Eq. (1), where *χ*_1_ is the temperature, *χ*_2_ is the ISC, and *χ*_3_ is the C/N ratio.

(1) $$\begin{array}{c} \text{Biogas}\text{yield}\left(\text{mL}{\text{gVS}}^{-1}\right)=-2215+99.96\chi_{1}+38.59\chi_{2}-35.27\chi_{3}+0.4188\chi_{1}\chi_{2}+0.6294\chi_{1}\chi_{3}+0.6942\chi_{2}\chi_{3}-1.281{\chi_{1}}^{2}-1.487{\chi_{2}}^{2}-0.4046{\chi_{3}}^{2}. \end{array}$$

ANOVA was conducted for the response variable, as presented in Table 4. The model *F* value of 56.10 indicated that the quadratic model was statistically significant for biogas production. The high *R*^2^ coefficient of 0.9883 ensured a satisfactory adjustment of the quadratic model to the experimental data, implying that only 1.17% of biogas production variability could not be explained by the proposed model. The adjusted *R*-square (*R*_adj_^2^ = 0.9706) and Adeq-Precision of 18.17 were also high, supporting the high significance of the model. The lack-of-fit *F* value of 0.33 revealed that the lack of fit was insignificant. Therefore, it is reasonable to believe that the proposed model is reliable for predicting the biogas production of SS-AD.

The significance test for the regression coefficients determined by *p* values was carried out. The larger the *F* value of the regression parameter was, the smaller the *p* values were, indicating that this parameter has a greater impact on biogas production. The corresponding *p* values showed that all the factors, including interactive terms, were significant model terms, except the ISC (*F*value = 2.71, *p*value = 0.1510). Among the three operating parameters, the C/N ratio (*F*value = 68.04, *p*value = 0.002) had the largest effect on biogas production, followed by temperature (*F*value = 17.01, *p*value = 0.0062).

**Figure 1:**
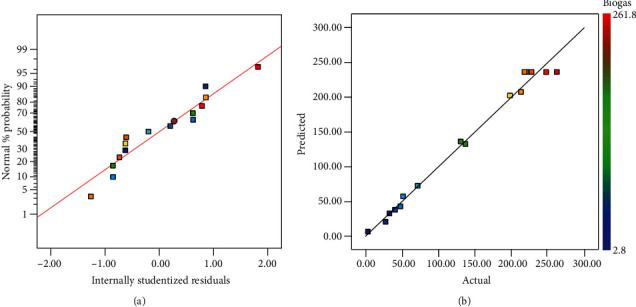
Plots of standardized residuals vs. normal % probability for biogas production (a). Actual and predicted values of biogas production (b).

It is important to check the model adequacy of biogas production for response surface optimization, as the model would give poor or misleading results if not well fit. The normal probability plot indicates whether the residuals follow a normal distribution, in which case the points will follow a straight line [29]. The normality assumption was examined by constructing a plot of standardized residuals vs. the normal % probability. As shown in Figure 1(a), the normal probability plot was approximated well by a straight line, indicating that the response variables did not require transformation, and that there were no apparent abnormality issues [29].  Figure 1(b) shows that the predicted values of the responses from the biogas production model accorded well with the actual values. The distribution of data plots is relatively close to a straight line, indicating reasonably adequate agreement between the actual and predicted values and confirming that the model could be further used to navigate the space defined by BBD.

### 3.4. Optimization of Biogas Production Operating Parameters

**Figure 2:**
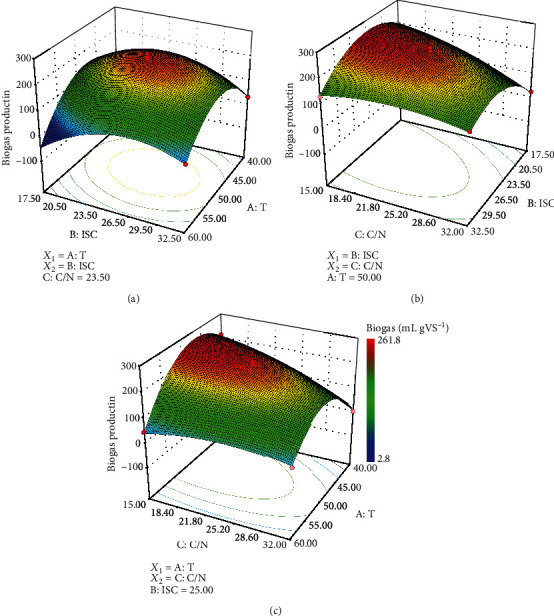
Effects of the ISC, T, and C/N ratio on biogas production. (a) Interactive effect of the ISC and T at a C/N ratio of 23.5. (b) Interactive effect of the ISC and C/N ratio at 50°C. (c) Interactive effect of the T and C/N ratio at an ISC of 25%.

The experimental results were visualized in three-dimensional response surface plots and the corresponding contour plot, which show the simultaneous effect of two independent factors on biogas production, with one variable maintained at its central level.  Figure 2(a) illustrates the effects of ISC and T on biogas production at a C/N ratio of 23.5. Noticeable changes in the biogas production by changing each parameter were recorded, and the results showed that both parameters and their interaction are effective [8]. According to Figure 2(a), biogas production showed a significant increasing trend as the ISC increased from 17.5 to 25% and temperature increased from 40 to 50°C; the biogas production then decreased when the ISC and temperature increased beyond those values.

The TS content is responsible for the mass transfer in SS-AD [30]. It is believed that a decreasing TS content might facilitate substrate conversion in SS-AD because metabolism by microorganisms (including hydrolytic bacteria, acidogenic bacteria, acetogenic bacteria, and methanogenic archaea) occurs in the water-soluble phase [31–33]. However, the maximum value of biogas production was not achieved at the lowest ISC (17.5%) in this study. It appears that in this study, the increase in the feed TS content up to 25% has a positive effect on biogas production. Yi et al. [34] showed that biogas production increased as the TS content increased from 5%-20%, and Paritosh et al. [35] reported that increasing the TS content beyond 25% did not result in a significant increase in the methane yield, consistent with our results. Yan et al. [8] reported that biogas production decreased drastically when increasing the ISC from 20 to 35%. This phenomenon suggested that the effect of the TS content on biogas production is related to the specific characteristics of the substrates, the type of inoculum, and the interaction of other operating parameters (such as process temperature) [30]. The operating temperature determines the fate of the microbes, which may disturb the overall reaction process. In this study, further increasing the temperature above 50°C exhibited a negative effect on biogas production. It has been reported that thermophilic conditions in SS-AD enhance the hydrolysis of the substrate by stimulating hydrolytic microorganisms, and an excessive process temperature may hamper or inhibit the methanogenesis process due to VFA accumulation [30, 36]. The results from previous studies were consistent with our results.

Figures 2(b) and 2(c) illustrate the effects of the ISC and C/N ratio on biogas production at 50°C and the effects of the T and C/N ratio on biogas production at an ISC of 25%, respectively. It is found that the enhancement of biogas production can be achieved when the ISC increased from 17.5 to 25% at the temperature of 50°C and then decreased gradually after that (Figure 2(b)), showing no difference from the results shown in Figure 2(a). It is also observed that the biogas production decreased when the C/N ratio increased from 15 to 32 at the temperature of 50°C, confirming that a high C/N ratio results in the rapid consumption of nitrogen by methanogens and further results in a lower biogas yield [37]. These results also suggested that the microbial activities for hydrolysis and methanogenesis at 50°C could withstand the inhibition caused by excessive free ammonia production.

### 3.5. Optimization of SS-AD

The SS-AD parameters were optimized based on the quadratic model using the optimization module of the Design Expert software. The results showed that a maximum biogas production capacity of 265 mL gVS^−1^ would be achieved with an ISC of 23.4%, T of 47.7°C, and C/N ratio of 17.2, without considering other factors. It is noteworthy that the optimized C/N ratio is relatively low, indicating that considerable extra urea or other nitrogen sources should be added during the SS-AD process, which would increase the operating cost. Moreover, a low C/N ratio might hinder the composting of the digestate of SS-AD [38]. Torres-Climent et al. reported that a C/N ratio of 28-31 showed the most rapid temperature increase during composting of the solid phase of digestate [39]. Additionally, a relatively lower temperature would be desired for SS-AD because of the stability and lower heating energy costs. Therefore, a goal was set to maximize biogas production and the C/N ratio while minimizing the process temperature. Concretely, for optimization, the importance of temperature and the C/N ratio were considered important (+), and biogas production was considered the most important (+++++). The results indicated that a biogas productivity of 241.4 mL gVS^−1^ would be achieved at 47.3°C, ISC = 24.81%, and C/N = 22.35. As the optimized parameters, i.e., ISC, C/N ratio, and T, are close to the parameters designed in this study, a verification experiment of SS-AD was not further carried out.

### 3.6. Comparison of the Community Diversity of Bacteria

**Figure 3:**
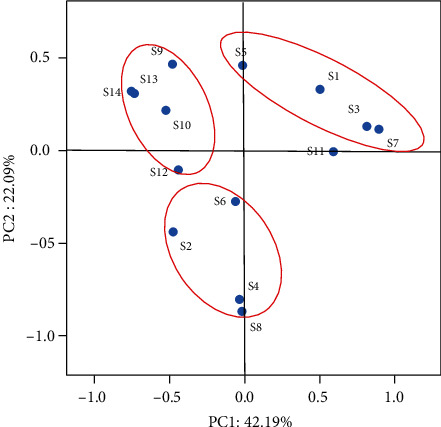
PCoA plot comparing bacterial communities from different 20 d SS-AD samples.

**Figure 4:**
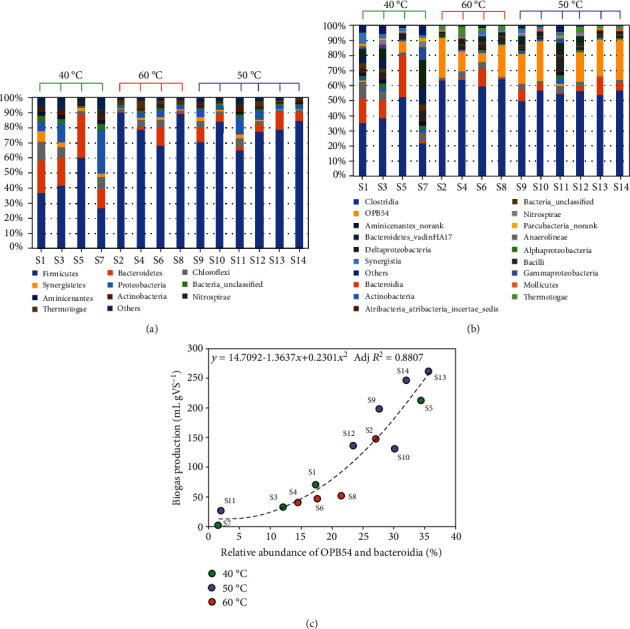
Major bacterial phyla (each represented by >1% total sequences in at least one sample) (a), relative abundance (% of the total bacterial sequences in each sample) of the major OTUs (b), and relationship between biogas production and relative abundance of *OPB54* and *Bacteroidia* (c) after 20 d of SS-AD.

After 20 d, the bacterial communities in SS-AD samples from run 1 to run 14 were further characterized by amplicon sequencing of 16S rRNA genes. As shown in Table 5, different high-quality sequences ranging from 16,877 to 33,006 per sample were obtained. All of the sequences were aligned and clustered to calculate the operational taxonomic units (OTUs) based on 97% sequence identity, resulting in 1105 OTUs at a sequencing depth of 16000 reads per sample and coverage of 99.0-99.4%. These results showed that the Chao 1 estimator of richness and Shannon diversity index of the microbial community at 60°C (except S6) were lower than those at 50°C, indicating that the microbial communities at 50°C were generally more complex than those at 60°C. The differences in microbial communities among SS-AD samples were also described by principal coordinate analysis (PCoA), as shown in Figure 3. The first two axes of the PCoA explained 42.19% and 22.09% of the variance, respectively, or collectively 64.28% of the variance. This analysis also showed a distinct community structure among SS-AD samples that can be separated into three groups based on incubation temperatures (i.e., 40, 50, and 60°C), except S11, which had excessive acidification (Figure 4).

Ten major phyla, represented by more than 1% of the total bacterial sequence of each phylum, were found for the SS-AD samples. This result is not surprising because distinct differences in the relative abundance of these phyla were observed with various operating parameters. *Firmicutes* and *Bacteroidetes* have been repeatedly reported as the main phyla in different anaerobic digesters [40]. In this study, *Firmicutes* sequences indicated that it was the most predominant phylum at temperatures of 50°C and 60°C, accounting for 65-90% of the total bacterial sequences; the percentages of *Firmicutes* sequences in the SS-AD samples were 37.5-60% at 40°C, except for S7 (27%). *Bacteroidetes* was the second most prevalent phylum at 40°C (18.62-28.37%, except for S7), but it only represented 0.76-12.21% of the total bacterial sequences at 50 and 60°C. These observations of a higher abundance of *Firmicutes* and a lower abundance of *Bacteroidetes* at the process temperatures of 50-60°C compared with 40°C are consistent with the results from previous studies [41, 42], indicating a competitive advantage of *Firmicutes* over *Bacteroidetes* at elevated temperatures [40]. For the phylum *Thermotogae*, the percentage of sequences at 60°C (2.07-5.45%) is much higher than that at other temperatures (0.15-1.29%), except for S12 (2.85%). Due to the SS-AD failure, the major bacterial phyla of S7 were dramatically different from those of other samples at 40°C (Figure 4(a)), containing a lower percentage of *Firmicutes* sequences and *Bacteroidetes* sequences and a much higher percentage of *Proteobacteria* sequences. It also noted that S11, with excessive acidification, contained a higher percentage of *Proteobacteria*, *Aminicenantes*, *Actinobacteria*, and *Nitrospirae* sequences and a lower percentage of *Firmicutes* sequences than the other samples at 50°C.

A total of 18 classes were identified for SS-AD samples that harboured ≥1% of the reads in one or more of the sequences, while *Clostridia*, *Bacteroidia*, *Anaerolineae*, and *OPB54* were the primary communities (Figure 4(b)). Among them, the class *Clostridia* has been associated with hydrolysis, acidogenesis, and acetogenesis steps [40]. *OPB54* was previously identified in an enrichment culture of lignocellulosic biomass and was the most abundant anaerobic syntrophic acetate-oxidizing genus in biogas digesters fed with high levels of acetate [43, 44]. The difference between the S13 and S14 samples, which have identical operating parameters, was quite small at the class level, where *OPB54_norank* in the phylum *Firmicutes* was the most dominant genus (Figure S2). It is worth noting that the biogas production values and relative abundances of *OPB54* and *Bacteroidia* after 20 d of SS-AD can be fitted well using a quadratic model (Figure 4(c)), with Adj*R*^2^ = 0.8807, implying that *OPB54* and *Bacteroidia* play important roles in the methanogenic metabolism for agriculture waste thermophilic SS-AD.

## 4. Conclusion

Optimizing the process parameters, i.e., temperature (40-60°C), initial solid content (17.5-32.5%), and C/N ratio (15-32 : 1), is important to promote the thermophilic SS-AD performance of the agriculture waste. All the factors, including interactive terms (except the ISC), were significant in the quadratic model for biogas production. Among the three operating parameters, the C/N ratio had the largest effect on biogas production, followed by temperature, and the maximum biogas yield of 241.4 mL gVS^−1^ could be achieved at 47.3°C, ISC = 24.81%, and C/N = 22.35. After 20 d of SS-AD, PCoA showed a distinct community structure that could be separated into three groups based on incubation temperatures, except for the SS-AD sample with excessive acidification. The biogas production values and relative abundance of *OPB54* and *Bacteroidia* after 20 d of SS-AD can be fitted well using a quadratic model, implying that *OPB54* and *Bacteroidia* play important roles in the methanogenic metabolism.

## Figures and Tables

**Table 1:** Fundamental characteristics of corn straw, cattle manure, and vegetable waste.

Type	C (%)^∗^	N (%)^∗^	C/N	TS (%)	VS (%)	pH
Corn straw	43.36	1.26	34.49	88.16	87.31	6.69
Cattle manure	37.34	2.55	14.67	83.28	78.83	8.56
Vegetable waste	34.37	4.68	7.34	3.42	89.14	7.46

^∗^Total carbon and nitrogen (dry basis) were determined by an elemental CHN analyser (Euro EA3000, Euro Vector, Italy).

**Table 2:** Three-level-three-factor Box–Behnken design for SS-AD of corn straw, cattle manure, and vegetable waste.

Run	*χ*_1_	*χ*_2_	*χ*_3_	Mixed substrate^∗^	Inoculum^#^	Distilled water	Urea
	A : T	B : ISC	C : C/N	(g)	(g)	(mL)	(g)
1	40	17.5	23.5	100	137.02	86	0.345
2	60	17.5	23.5	100	137.02	86	0.345
3	40	32.5	23.5	100	137.02	0	0.345
4	60	32.5	23.5	100	137.02	0	0.345
5	40	25	15	100	137.02	30	1.091
6	60	25	15	100	137.02	30	1.091
7	40	25	32	100	137.02	30	0.000
8	60	25	32	100	137.02	30	0.000
9	50	17.5	15	100	137.02	86	1.091
10	50	32.5	15	100	137.02	0	1.091
11	50	17.5	32	100	137.02	86	0
12	50	32.5	32	100	137.02	0	0
13	50	25	23.5	100	137.02	30	0.345
14	50	25	23.5	100	137.02	30	0.345
15	50	25	23.5	100	137.02	30	0.345
16	50	25	23.5	100	137.02	30	0.345
17	50	25	23.5	100	137.02	30	0.345

^∗^With a moisture content of 67.5%, the mass ratio of cornstraw/cattlemanure/vegetablewaste = 40 : 10 : 0.5 (dry wt.). ^#^The feedstock-to-inoculum ratio (F/I, based on VS) was 3.

**Table 3:** Effect of T, ISC, and C/N ratio on the biogas yield, pH, and TAN concentration after 20 d of SS-AD.

Run	*χ*_1_	*χ*_2_	*χ*_3_	Biogas yield (mL gVS^−1^)	pH	TAN concentration (mg kg^−1^)
	A : T	B : ISC	C : C/N			
1	40	17.5	23.5	71.12	8.46	1640.7
2	60	17.5	23.5	147.2	8.67	1807.1
3	40	32.5	23.5	32.60	8.02	2437.1
4	60	32.5	23.5	40.12	8.16	3281.9
5	40	25	15	212.9	8.89	1889.6
6	60	25	15	47.42	8.41	3613.4
7	40	25	32	2.80	7.49	1925.9
8	60	25	32	51.31	8.13	2066.2
9	50	17.5	15	198.1	8.69	2275.4
10	50	32.5	15	130.7	8.85	3367.3
11	50	17.5	32	26.89	6.06	1836.3
12	50	32.5	32	136.5	8.78	2343.7
13	50	25	23.5	261.8	8.67	1888.4
14	50	25	23.5	247.1	8.70	
15	50	25	23.5	225.5	8.64	
16	50	25	23.5	217.9	8.61	
17	50	25	23.5	227.2	8.57	

**Table 4:** ANOVA for the quadratic model of SS-AD biogas production.

Source	Sum of squares	df	Mean square	*F* value	*p* value	
Model	128108	9	14234	56.10	<0.0001	Significant
*χ*_1_-T	4315	1	4315	17.01	0.0062	
*χ*_2_-ISC	687	1	687	2.71	0.1510	
*χ*_3_-C/N	17263	1	17263	68.04	0.0002	
*χ*_1__,_ *χ*_2_	1973	1	1973	7.78	0.0316	
*χ*_1__,_ *χ*_3_	11448	1	11448	45.12	0.0005	
*χ*_2__,_ *χ*_3_	7833	1	7833	30.87	0.0014	
*χ*_1_^2^	54668	1	54668	215.46	<0.0001	
*χ*_2_^2^	23310	1	23310	91.87	<0.0001	
*χ*_3_^2^	2848	1	2848	11.22	0.0154	
Residual	1522	6	254			
Lack of fit	218	2	109	0.33	0.7338	Not significant
Pure error	1304	4	326			
Cor total	129630	15				
*R* ^2^ = 0.9883; Adj *R*^2^ = 0.9706; Adeqprecision = 18.17						

**Table 5:** Microbial diversity indices based on 97% identity of 16S rRNA gene sequences.

Sample	Sequences	Observed species	Chao 1	Shannon index	Coverage
S1	27252	777.8	773.74	4.84	99.10%
S2	26466	607.56	641.6	3.8	99.10%
S3	31804	731.53	739.76	4.67	99.10%
S4	16963	636.68	660.15	4.14	99.10%
S5	16877	627.73	609.84	4.31	99.20%
S6	33006	812.68	805.12	4.73	99.00%
S7	20373	615.82	608.88	4.52	99.40%
S8	27445	784.3	671.71	3.87	99.00%
S9	25602	767.25	763.11	4.77	99.10%
S10	24824	709.31	705.33	4.32	99.00%
S11	27304	699.89	712.33	4.75	99.20%
S12	17586	704.69	720.62	4.42	99.10%
S13	24440	680.86	720.56	4.19	99.10%
S14	25193	621.92	623.55	4.17	99.20%

## Data Availability

The data used to support the findings of this study are available from the corresponding author upon request.

## Supplementary Materials

Supplementary materials Figure S1: SS-AD reactor used in this study. Figure S2: major bacterial genera (a list of the 50 most abundant genera) after 20 d of SS-AD.
