# Supplementary material for: Thermophilic Solid-State Anaerobic Digestion of Corn Straw, Cattle Manure, and Vegetable Waste: Effect of Temperature, Total Solid Content, and C/N Ratio
Source: Archaea. 2020 Nov 11;2020:8841490. doi: 10.1155/2020/8841490 (PMC7673934; doi:10.1155/2020/8841490)
Supplement: Supplementary materials — Figure S1: SS-AD reactor used in this study. Figure S2: major bacterial genera (a list of the 50 most abundant genera) after 20 d of SS-AD. [file 8841490.f1.docx]

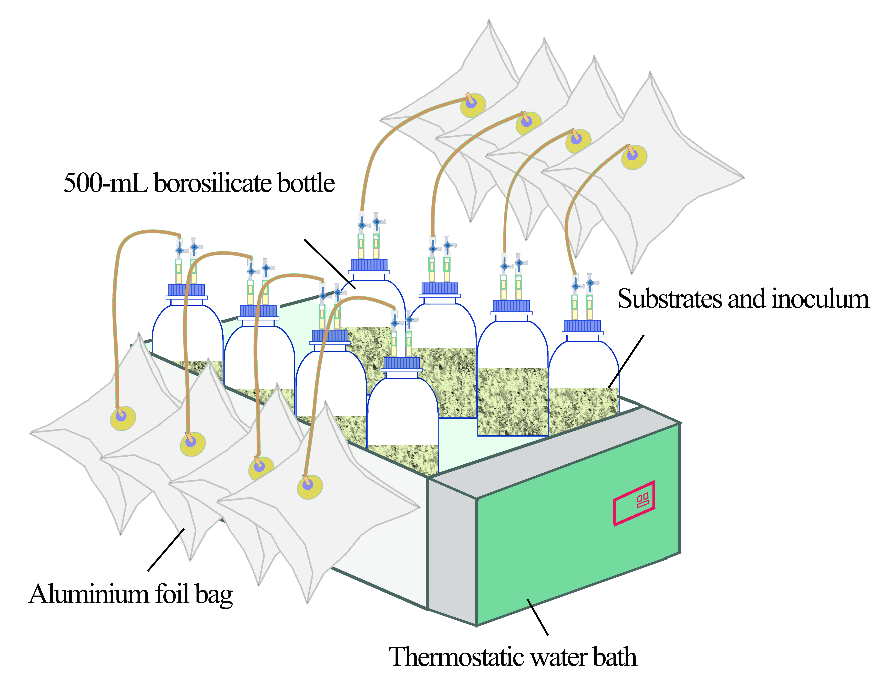


**Fig. S1** SS-AD reactor used in this study.


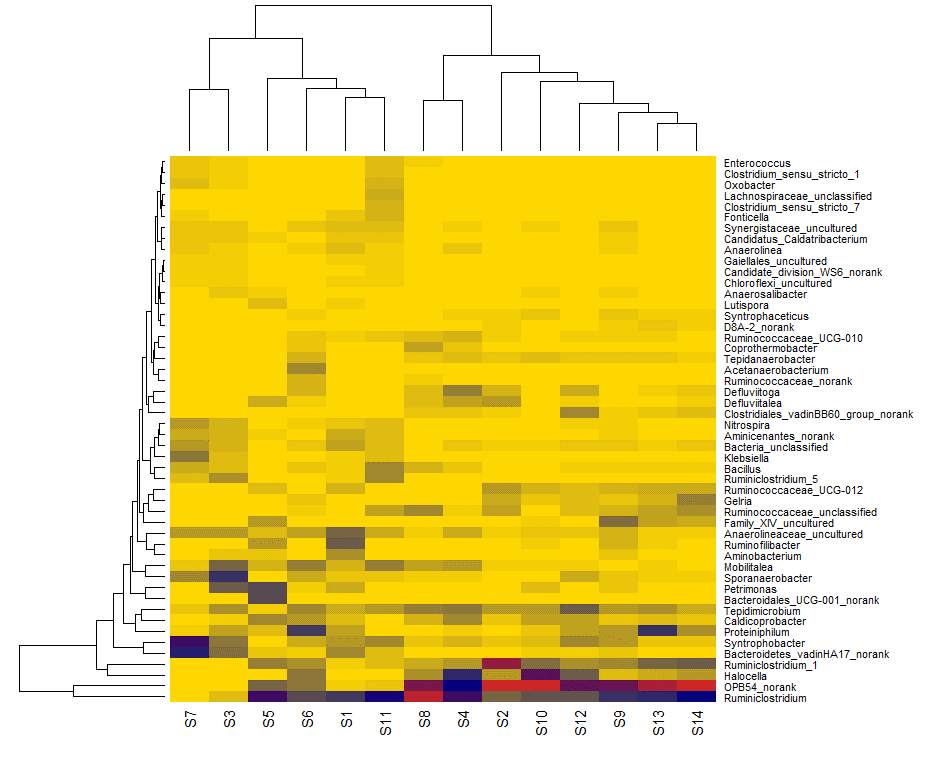


**Fig. S2** Major bacterial genera (a list of the 50 most abundant genera) after 20 d of SS-AD.
